# Sequence-Based Mapping and Genome Editing Reveal Mutations in Stickleback *Hps5* Cause Oculocutaneous Albinism and the *casper* Phenotype

**DOI:** 10.1534/g3.117.1125

**Published:** 2017-07-26

**Authors:** James C. Hart, Craig T. Miller

**Affiliations:** Department of Molecular and Cell Biology, University of California-Berkeley, California 94720

**Keywords:** genome editing, stickleback, pigmentation, albinism, Hermansky-Pudlak syndrome

## Abstract

Here, we present and characterize the spontaneous X-linked recessive mutation *casper*, which causes oculocutaneous albinism in threespine sticklebacks (*Gasterosteus aculeatus*). In humans, Hermansky-Pudlak syndrome results in pigmentation defects due to disrupted formation of the melanin-containing lysosomal-related organelle (LRO), the melanosome. *casper* mutants display not only reduced pigmentation of melanosomes in melanophores, but also reductions in the iridescent silver color from iridophores, while the yellow pigmentation from xanthophores appears unaffected. We mapped *casper* using high-throughput sequencing of genomic DNA from bulked *casper* mutants to a region of the stickleback X chromosome (chromosome 19) near the stickleback ortholog of Hermansky-Pudlak syndrome 5 (*Hps5*). *casper* mutants have an insertion of a single nucleotide in the sixth exon of *Hps5*, predicted to generate an early frameshift. Genome editing using CRISPR/Cas9 induced lesions in *Hps5* and phenocopied the *casper* mutation. Injecting single or paired *Hps5* guide RNAs revealed higher incidences of genomic deletions from paired guide RNAs compared to single gRNAs. Stickleback *Hps5* provides a genetic system where a hemizygous locus in XY males and a diploid locus in XX females can be used to generate an easily scored visible phenotype, facilitating quantitative studies of different genome editing approaches. Lastly, we show the ability to better visualize patterns of fluorescent transgenic reporters in *Hps5* mutant fish. Thus, *Hps5* mutations present an opportunity to study pigmented LROs in the emerging stickleback model system, as well as a tool to aid in assaying genome editing and visualizing enhancer activity in transgenic fish.

The combination of new genome editing methods and next-generation sequencing technologies has ushered in a new era in genetics. High-throughput DNA sequencing enables rapid forward genetic mapping of Mendelian (Schneeberger *et al.* 2009; Cuperus *et al.* 2010; Doitsidou *et al.* 2010; Zuryn *et al.* 2010; Bowen *et al.* 2012; Obholzer *et al.* 2012) and quantitative (Glazer *et al.* 2014, 2015; Jamann *et al.* 2015) loci. The remarkably high efficiency of the CRISPR/Cas9 system across diverse species (Friedland *et al.* 2013; Hwang *et al.* 2013; Jinek *et al.* 2013; Guo *et al.* 2014; Square *et al.* 2015; Martin *et al.* 2016) now allows for functional reverse genetic analysis in any species competent for delivery of genome editing reagents. However, the optimization of genome editing protocols is still in its infancy, with the efficiency of DNA double-strand break formation and repair still requiring characterization in many species. Particularly powerful loci for this characterization are those that mutate to cause an obvious viable visible phenotype, such as loci that affect pigmentation (Dahlem *et al.* 2012; Guo *et al.* 2014; Irion *et al.* 2014; Square *et al.* 2015; Burger *et al.* 2016; Hoshijima *et al.* 2016).

The diversity of vertebrate skin pigmentation is due to an interplay between four main groups of pigment-containing cells known as chromatophores (Fujii 2000; Kelsh 2004; Mills and Patterson 2009; Irion *et al.* 2016). Chromatophores originate from the neural crest, which migrate and then differentiate into pigment cell types during embryonic development (Fujii 2000; Kelsh *et al.* 2009). Melanophores possess black or dark brown melanin-containing organelles, melanosomes, which are also present in the retinal pigment epithelium (RPE) (Marks and Seabra 2001; Wasmeier *et al.* 2008). Iridophores appear iridescent and typically silver due to the presence of light-reflective guanine-containing platelets, the size and orientation of which determine the reflective color (Cooper *et al.* 1990; Oshima 2001). Xanthophores appear yellow-red due to the presence of pteridine within pterinosomes (Ziegler 2003). Erythrophores contain vesicles with red carotenoids obtained through the diet, and are a critical mating cue in sticklebacks (Milinski and Bakker 1990; Wedekind *et al.* 1998; Fujii 2000). Pigment cells in insects also possess melanin-, ommochrome-, or drosopterin-containing organelles (Shoup 1966).

Pigment-containing organelles belong to a larger class of cellular organelles, the lysosome-related organelles (LROs) (Dell’Angelica *et al.* 2000; Dell’Angelica 2004). LROs encompass a wide variety of organelles in different cellular contexts, including platelet granules, basophil granules, neutrophil azurophil granules, major histocompatibility complex class II compartments, lamellar bodies, osteoclast granules, and lytic granules (Dell’Angelica *et al.* 2000; Marks and Seabra 2001; Dell’Angelica 2004). The best studied LRO, the melanosome, has a well characterized biogenesis. Starting at stage I, premelanosomes already have internal vesicles and interluminal fibers that become parallel and organized during stage II, darkening during stage III until they are obscured by melanin in fully formed stage IV melanosomes (Marks and Seabra 2001). Patients with melanosome maturation defects often exhibit defects in other LROs, revealing that different LROs share similar biogenesis pathways (Marks and Seabra 2001).

In a wide range of vertebrates and invertebrates, a highly conserved set of genes including members of biogenesis of LRO complexes (BLOC) 1, 2, and 3 (Helip-Wooley *et al.* 2007; Wei *et al.* 2013) regulate the formation and maturation of LROs, including pigment producing LROs, and are required for wild-type pigmentation (Helip-Wooley *et al.* 2007; Wei *et al.* 2013). In humans, mutations in the BLOC-2 complex member *Hps5* result in Hermansky-Pudlak Syndrome type 5, which is characterized by oculocutaneous albinism, and bleeding diathesis (Huizing *et al.* 2004). Mutations in orthologs of *Hps5* result in oculocutaneous albinism in a wide variety of model organisms, including the *ruby-eyed 2* phenotype in mice (Zhang *et al.* 2003), the *snow white* phenotype in zebrafish (Daly *et al.* 2013), the *pink* phenotype in *Drosophila* (Falcón-Pérez *et al.* 2007; Syrzycka *et al.* 2007), and the *translucent* phenotype in silkworms (Fujii *et al.* 2012). In vertebrates, Hps5 is required for the maturation of type I to type II melanosomes (Nguyen *et al.* 2002), and also binds to and stabilizes other HPS proteins including Hps3 and Hps6 (Daly *et al.* 2013). In addition to conserved roles in melanosome maturation, *Hps5* and *Hps6* are required for iridophore development in zebrafish and *Xenopus*, respectively (Daly *et al.* 2013; Nakayama *et al.* 2016).

In zebrafish, the *snow white* mutant phenotype, oculocutaneous albinism, was shown to result from an I76N point mutation in the WD40 domain of Hps5 (Daly *et al.* 2013). This mutation results in relatively mild reductions in RPE and melanophore melanization due to fewer and smaller melanosomes, and loss of iridophores. *snow white* mutants display early larval lethality, representing the only lethal *Hps5* allele reported, as well as the only mutation within the N-terminal WD40 domain (Daly *et al.* 2013).

Here, we report the discovery and characterization of a spontaneous threespine stickleback X-linked recessive albino mutation *casper*. A mapping-by-sequencing approach revealed *casper* to be tightly linked to the stickleback ortholog of *Hps5*. *casper* mutants have a 1 bp insertion, resulting in a predicted frame-shift mutation in *Hps5* that results in an early truncation of the highly conserved protein product. Inducing mutations in *Hps5* using the CRISPR/Cas9 system phenocopied the *casper* mutation. Lastly, we show the usefulness of *casper* embryos as a tool to visualize fluorescent reporters in adult fish. Together these data provide a new locus in an emergent genetic supermodel (Gibson 2005) to facilitate studies of genome editing, transgene expression, and pigmentation biology.

## Materials and Methods

### Animal husbandry and imaging

Fish were raised in brackish water (3.5 g/L Instant Ocean salt, 0.217 ml/L 10% sodium bicarbonate) at 18° in 8 hr of light per day in 110 L aquaria. Fry with standard length (SL) less than ∼10 mm were fed a diet of live *Artemia*, with frozen *Daphnia* added as fish reached ∼10 mm SL. Adults with SL greater than ∼20 mm were fed a combination of frozen bloodworms and *Mysis* shrimp. To map the *casper* mutation, six crosses were generated by crossing four different marine males to heterozygous *casper* mutant females (Supplemental Material, Table S1). Experiments were approved by the Institutional Animal Care and Use Committees of the University of California-Berkeley (protocol # AUP-2015-01-7117). Embryos were visualized using Montage z-stacks on a Leica M165 FC dissecting microscope, using a GFP2 filter to visualize xanthophores, or on a Keyence VHX-5000 microscope. Adult fish were imaged using a Cannon Powershot S95 digital camera.

### DNA purification and sequencing

For sequencing and genotyping, DNA was extracted from caudal fin tissue from the original *casper* male, the F_0_ female he was crossed to, as well as 47 individual *casper* mutant F_2_ embryos. Caudal fin tissue or embryos were digested for 12 hr at 55° in 600 μl of tail digestion buffer [10 mM Tris pH 8.0, 100 mM NaCl, 10 mM EDTA, 0.05% SDS, 2.5 μl Proteinase K (Ambion AM2546)].

DNA from whole *casper* mutant F_2_ embryos was diluted to ∼10 ng/μl for each fish and pooled. Barcoded Nextera libraries (Illumina FC-121-1031) were created from 50 ng of genomic DNA from the pooled embryos, as well as 50 ng of genomic DNA from the original *casper* male and F_0_ female mate following the manufacturer’s instructions. Quality was assayed on an Agilent bioanalyzer, and the resulting libraries were sequenced on a single lane of an Illumina HiSeq4000 to generate 100 bp single-end reads.

### Bulk segregant analysis

Reads were mapped to a revised assembly of the stickleback genome (Jones *et al.* 2012; Glazer *et al.* 2015), using bowtie2 (Langmead and Salzberg 2012) with parameters “-q–sensitive” (Table S2). Resulting SAM files were converted to BAM files, and sorted using Samtools version 0.1.18 (Li *et al.* 2009). Read groups were added, CIGAR strings fixed, mate pair information was fixed, and PCR duplicates were removed using picard tools v 1.51 (http://broadinstitute.github.io/picard). The Genome Analysis Tool Kit (McKenna *et al.* 2010; DePristo *et al.* 2011; Van der Auwera *et al.* 2013) (GATK)’s (v3.2-2) IndelRealigner (parameter: “-LOD 0.4”), BaseRecalibrator, and PrintReads were used to finalize preprocessing of BAM files. Finally, Unified Genotyper was used to call variants, with parameters “–genotype_likelihoods_model BOTH -stand_call_conf 50.” The resulting VCF file was filtered for variants which had qual score >40, had more than five reads covering the variant, and were not found in the F_0_ female mated to the original *casper* male. Analysis was performed using a custom ipython notebook (see *Data availability* section below for link to custom scripts). Briefly, we computed the proportion of each variant that matched the *casper* allele, and results were smoothed by plotting the proportion of *casper* reads within a 50 variant sliding window, advancing five variants at a time. As we found *casper* to be X-linked, we reasoned that the hemizygous XY *casper* animals should all be identical in sequence around the causative locus. We computed, as an additional measure, the proportion of variants within the window with reads matching more than one allele, which is expected to be 0 at the causative locus.

### PCR, cloning, and Sanger sequencing validation

PCR primers (Table S3) were designed using Primer3 (Rozen and Skaletsky 2000) and ordered from IDT. PCR was performed using Phusion DNA polymerase (Macro laboratory, University of California [UC]-Berkeley) and Phusion Buffer (NEB B0518S). To obtain sequence of single clones, PCR products were purified using a Qiagen PCR purification kit, and digested with *Xho*I (NEB R0146L) and *Xba*I (NEB R0145L) in cutsmart buffer for 1 hr at 37°. Digested products were ligated into a pBluescript II SK+ vector cut with *Xba*I and *Xho*I, transformed, and plated onto LB agar plates with ampicillin, IPTG, and X-gal. White colonies were picked and used as input to PCR as described above, adding a 5 min incubation at 95° before thermocycling. Resulting reactions were purified using a Qiagen PCR purification kit. Purified PCR product (∼20 ng) was Sanger sequenced by the UC-Berkeley DNA sequencing facility and results visualized using abiview.

### Genome editing of Hps5

Genome editing reagents were designed as previously described (Talbot and Amacher 2014). Briefly, pCS2-nCas9n (Addgene 7929) was linearized following digestion with *Not*I. Linearized plasmid (∼600 ng) was used as input to the mMessage SP6 kit, following the manufacturer’s instructions. mRNA quality was verified by running 0.5 μl of the reaction in 0.1%SDS on a Tris acetic acid EDTA (TAE) gel.

Guide RNAs (gRNAs) were designed using ZiFiT (Sander *et al.* 2010). DNA oligos were ordered from IDT, and gRNA templates were created with T7 promoters by PCR using Phusion polymerase. Resulting PCR products were gel purified, and 100 ng of the resulting elution was used as input to the MAXIscript T7 kit (Ambion), and guide RNA quality verified by running 0.5 μl of the reaction in a 50% formamide buffer on a TAE gel. Resulting gRNAs were precipitated using lithium chloride, incubated at −80° following addition of 75 μl 100% ethanol, and centrifuged at 15,000 rpm for 1 hr at 4°. Following a wash with 200 μl of 75% ethanol and an additional centrifugation at 15,000 rpm for 10 min at 4°, RNAs were resuspended in 20 μl of DEPC-treated water.

Stickleback embryos at the one cell stage were microinjected as described (Erickson *et al.* 2016) with some modifications. First, the concentration of gRNA in the 0.2 M KCl injection mixture was increased to 50 ng/μl. The Cas9 mRNA concentration was also doubled to 160 ng/μl, with 0.025% phenol red used as a tracking dye. Embryos were scored for *casper*-like phenotypes at 4 d postfertilization (dpf). Embryos were scored by eye, with embryos with any sign of mosaic albinism in their RPE classified as “mosaic,” and embryos with ∼75% albino RPE classified as “severe.” DNA from uninjected, wild-type injected, mosaic injected, and *casper*-like injected embryos was purified at 4–6 dpf, and deletions in *Hps5* were validated using Sanger sequencing of PCR products as described above.

### Data availability

Scripts used for bulk segregant analysis are available at https://github.com/trahsemaj/CASPER. The original *casper* male, the F_0_ mated to the *casper* male, and bulk segregant sequencing data are available from the SRA (accession number SRP111743).

## Results

### casper mutants display severely reduced pigmentation early during embryogenesis

**Figure 1:**
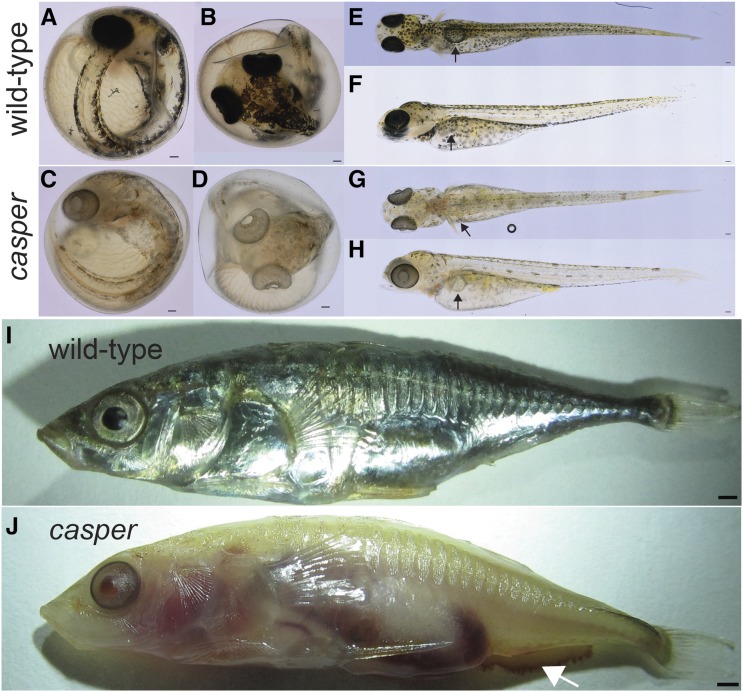
*casper* mutants display severely reduced eye and body pigmentation. (A–D) *casper* mutants are hypopigmented at 7 dpf and have severely reduced melanization of RPE: lateral (A, C) and dorsal (B, D) views. (E–H) Hypopigmentation of *casper* mutants persists at 14 dpf, with severely reduced melanophores but slightly melanized RPE: dorsal (E, G) and lateral (F, H) views. Black arrows indicate inflated swim bladder. (I, J) *casper* males are translucent at 5 months, with severely reduced iridophores and melanophore pigmentation, and highly reduced melanization of RPE. Mutants also bleed after euthanization (arrow in J). Bars, 100 μm (A–H), 1 mm (I).

We discovered a single spontaneous mutant male stickleback displaying severe pigmentation defects. We named this mutation *casper*, and recovered the mutation in subsequent generations (see below). *casper* mutants display oculocutaneous hypopigmentation in unhatched embryos when pigment first becomes apparent, becoming readily apparent by 7 dpf (Figure 1). Mutants appear fully viable and fertile (see below). *casper* mutants display severely reduced melanization of their RPE, the most obvious visible phenotype (Figure 1, A–D). Additionally, sexually mature *casper* males displayed severely reduced pigmentation in their testes relative to their wild-type siblings (Figure S1). Chromatophores are differentially affected in *casper* mutants. Beginning at the time of their first appearance (4 dpf), melanophores in *casper* mutants are present, but display severe reductions in melanization relative to their wild-type siblings (Figure 1, E–H). The silver pigmentation from iridophores appears absent from older *casper* fish (Figure 1, I and J). The red erythrophores, which contain diet-supplied carotenoids (Wedekind *et al.* 1998), were never observed in the throats of sexually mature *casper* mutant males. However, the yellow xanthophores (autofluorescent in sticklebacks) appear unaffected by the *casper* mutation in 10 dpf *casper* mutants (Figure S2).

*casper* mutants appear to initially inflate their swim bladder (Figure 1, E–H). Older mutants display variably penetrant minor difficulties in maintaining their position in the water column, suggesting possible swim bladder defects (14/15 1-month-old juvenile *casper* mutants were found within 1 cm of the bottom of their tank compared to 2/21 wild-type siblings, *P* < 0.01, Fisher’s exact test). Lastly, *casper* mutants display a bleeding phenotype, possibly due to a decreased clotting rate, following euthanization in 0.04% tricaine relative to their wild-type siblings (Figure 1, I–J).

### casper is a spontaneous, X-linked recessive albino mutation

**Figure 2:**
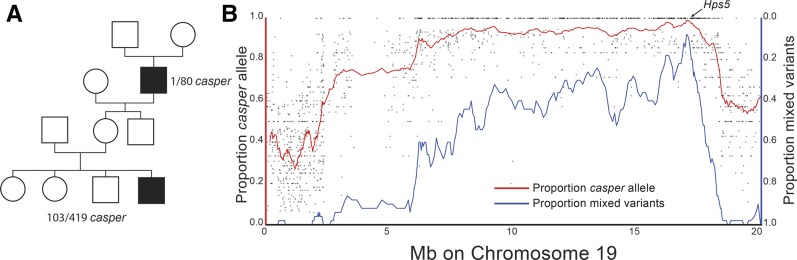
*casper* is X-linked and maps to a region of chromosome 19 near *Hps5*. (A) Pedigree of the spontaneous appearance of the original *casper* mutant and X-linked transmission in subsequent generations. Of the 103 *casper* mutants, 47 were genotyped, and all 47 were male by molecular genotyping with sex-specific primers. (B) Bulk segregant analysis of 47 *casper* mutants by high-throughput sequencing. Each point is the proportion of the variant allele with the *casper* genotype, with the red line showing a sliding window average across 50 variants. The blue line shows the proportion of variants called as heterozygous across a 50-variant sliding window. The peak of both red and blue lines is near the stickleback *Hps5* gene. *x*-axis shows the chromosome 19 revised genome assembly (Glazer *et al.* 2015). The top of the *y*-axis for the proportion of mixed variants (blue line) is zero.

The original *casper* mutant fish was a single male, and first appeared in a clutch of 80 fish from a cross between a marine male from the Rabbit Slough, Alaska population, and a freshwater creek female from the Cerrito Creek, California population (Figure 2A). To map the locus responsible for the *casper* phenotype, the original *casper* male was outcrossed to a female fish from a different marine population (Table S1). All the resulting F_1_ progeny were phenotypically wild-type, suggesting the *casper* mutation was either recessive or mosaic, with the germline of the original mutant fish not containing the *casper* mutation. As sticklebacks have a simple XY sex determination mechanism (Peichel *et al.* 2004), a spontaneous X-linked recessive mutation would be displayed in the original hemizygous male, but not in F_1_ progeny. We thus hypothesized that *casper* was X-linked. To test this hypothesis, we generated six outcrosses of F_1_ females to four males from two other populations and observed the F_2_s. Consistent with an X-linked mutation, 103/419 (24.6%) of the F_2_ offspring were *casper* mutants (Table S1), and molecular genotyping (Table S3) of 47 of the *casper* mutants showed that they were all male, confirming the sex-linked nature of *casper* (*P* < 1.4 × 10^−14^, two-tailed binomial test).

We next mapped the *casper* locus using a bulk segregant approach (Schneeberger *et al.* 2009; Cuperus *et al.* 2010; Doitsidou *et al.* 2010; Zuryn *et al.* 2010; Bowen *et al.* 2012; Obholzer *et al.* 2012). A barcoded Illumina sequencing library was created using genomic DNA pooled from 47 F_2_ *casper* males from six different F_1_ crosses. Additional barcoded libraries were created using DNA from the original *casper* fish as well as the F_0_ female crossed to *casper*, and all libraries were sequenced to moderate (∼10–14×) coverage (Table S2). We mapped *casper* by examining the proportion of reads at each variant position that matched the *casper* male’s allele. As an additional measure, we also looked for a loss of variants with mixed mapped reads (reflecting positions where most, or all, mutant male fish have the same X-chromosome genotype), as measured by the proportion of variants which only have reads supporting more than one allele within a 50 variant genomic window (Figure 2B). Both measures had similar peaks along stickleback chromosome 19, the stickleback X chromosome (Peichel *et al.* 2004), near the stickleback ortholog of a human oculocutaneous albinism gene, *Hermansky-Pudlak syndrome 5* (*Hps5*) (Figure 2B).

### casper is the result of the insertion of a single base-pair into the coding sequencing of Hps5

**Figure 3:**
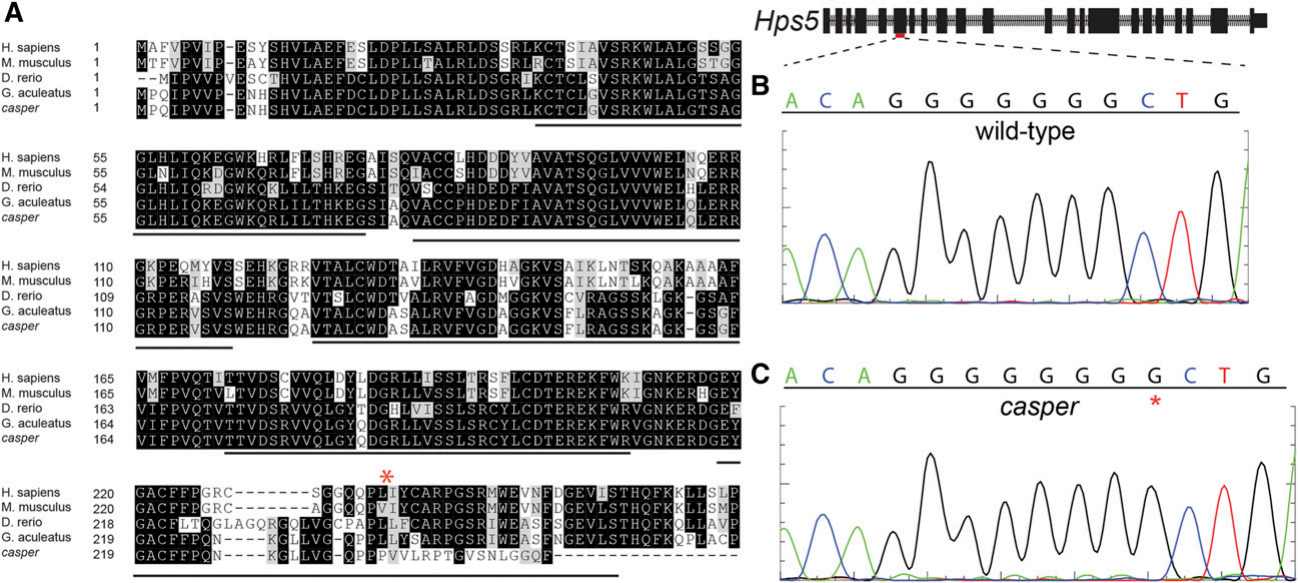
*casper* mutants contain a 1 bp insertion in exon 6 of *Hps5*. (A) A Clustal Omega (Sievers *et al.* 2011) multiple alignment of predicted amino-terminal HPS5 sequences from vertebrates with a known loss-of-function phenotype, as well as the predicted sequence from the original *casper* mutant (*casper*), and his wild-type F_0_ mate (*G. aculeatus*). Black lines indicate WD40 repeats predicted in human HPS5, and the red asterisk indicates the position of the *casper* insertion. See Figure S2 for full amino acid alignment. (B) Sanger sequencing of the red indicated region of *Hps5* in wild-type fish. (C) Sanger sequencing of the red indicated region of *Hps5* in the original *casper* mutant. The red “*” indicates the inserted G, which results in a predicted frame-shift and early truncation of the HPS5 protein.

We next sought to determine the mutation responsible for the *casper* phenotype. Genome-wide variant discovery using high-throughput sequencing data from the original *casper* male and the F_0_ wild-type female he was crossed to revealed 14 total variants within the predicted *Hps5* coding frame. Of these, 10 were synonymous point mutations, and two were substitutions found in both the affected *casper* male and unaffected female. The unaffected female had a mutation that results in a substitution from alanine to valine, which appears neutral (score of 0) in the BLOSUM62 matrix (Henikoff and Henikoff 1992), and which is also found in the orthologous mouse *Hps5* sequence (Figure 3A). The only remaining, and highest, impact variant was the insertion of a G in the sixth exon of *Hps5*, resulting in a frameshift and predicted early stop appearing seven codons following the novel insertion (Figure 3A and Figure S3). This variant is present only within the *casper* male, and not in his unaffected female mate. Sanger sequencing in both the wild-type female (Figure 3B) and *casper* male (Figure 3C) validated this insertion.

### Genome editing of Hps5 phenocopies casper

**Figure 4:**
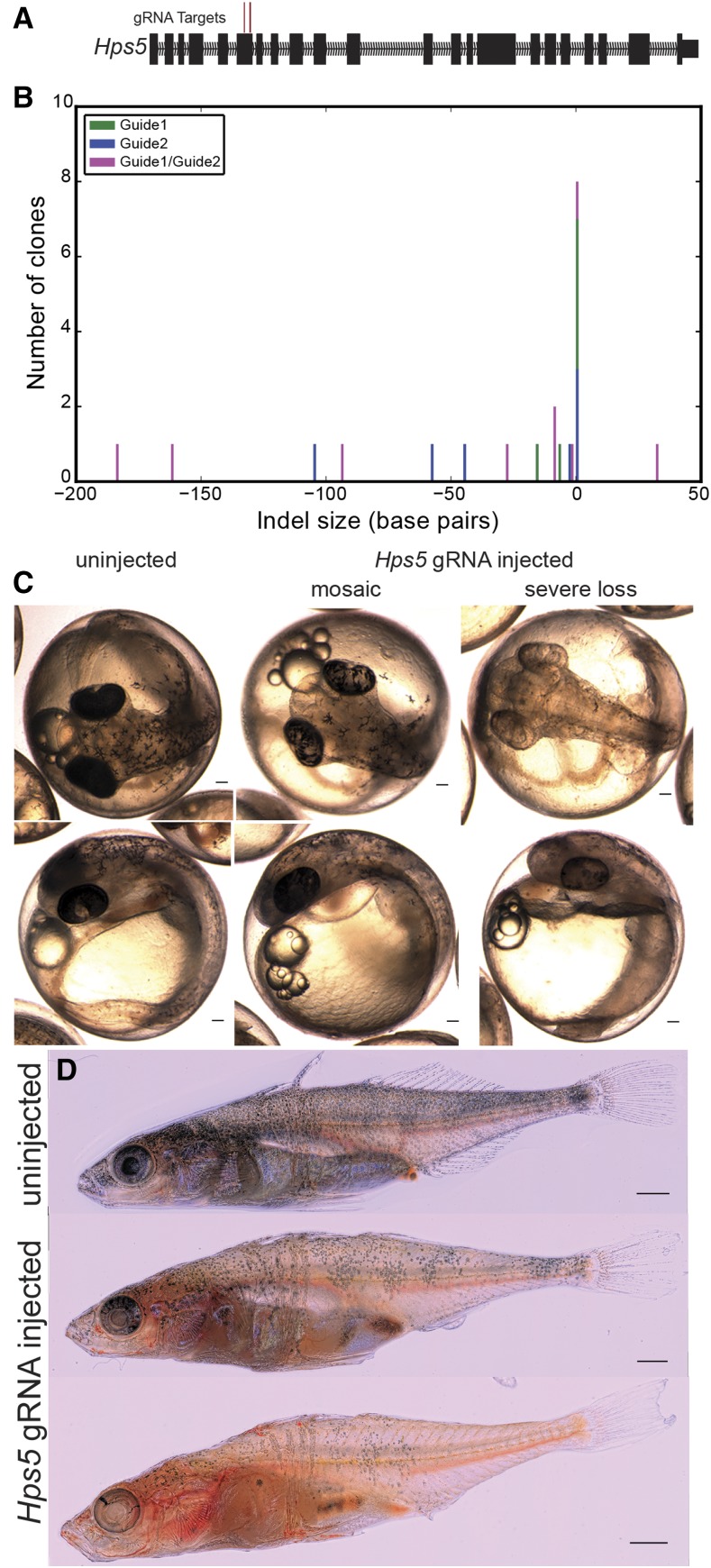
CRISPR/Cas9 induced mutations in *Hps5* phenocopy the *casper* mutation. (A) Two guide RNA sequences were targeted to the sixth exon of stickleback *Hps5* and were singly or coinjected along with Cas9 mRNA at the one-cell stage. (B) Sanger sequencing of clones derived from single representative *Hps5* injected fish reveals the highly efficient and mosaic nature of Cas9 mediated indel formation. (C) Most [29/32 (91%)] embryos injected with *Hps5* gRNAs displayed pigmentation reductions, with 14/32 (44%) displaying a mosaic loss of pigment in melanophores and RPE, and 15/32 (47% of F_0_ injected fish) displayed >75% loss of RPE (severe loss). (D) Adult *Hps5* gRNA injected sticklebacks show a mosaic loss of pigmented melanophores and iridophores, and are partially translucent. Bars, 100 μm (C), 1 mm (D).

We next tested whether other predicted loss-of-function mutations in stickleback *Hps5* could phenocopy *casper* mutants. The CRISPR/Cas9 system has been shown to be effective in genome editing in a wide range of model organisms, including another teleost, zebrafish (Hwang *et al.* 2013; Talbot and Amacher 2014). Two guide RNAs (gRNAs) were designed to target the sixth exon of stickleback *Hps5* (Figure 4A). Injection of either of the two gRNAs alone as well as Cas9 mRNA at the one-cell stage resulted in a wide range of insertions/deletions (indels) within the *Hps5* coding region of representative injected embryos (Figure 4B). Coinjection of the two gRNAs with Cas9 mRNA resulted in local indels around each gRNA target, but also larger deletions between the two, with an overall increase in indel size over single guides (*P* < 0.05, 1-tailed Mann-Whitney U, Figure 4B).

Hps5 gRNA injected embryos phenocopied *casper* mutants, with severely reduced melanization in both the RPE and melanophores (Figure 4C). Coinjection of these two gRNAs resulted in highly efficient induction of pigmentation phenotypes. Only 3/32 (9%) of the surviving embryos had fully wild-type pigmentation at 6 dpf, while 15/32 (46.9%) displayed a severe loss of pigment, and 14/32 (44%) appeared mosaic (Figure 4C). Embryos injected with only a single gRNA displayed a similar loss of pigmentation, though with decreased efficiency (*P* < 0.01, binomial test, Table S4). Overall, we observed severe or mosaic *casper*-like pigmentation phenotypes in 29/32 (91%) of injected embryos, and 8/9 (90%) of sequenced target regions contained indels near a protospacer adjacent motif (PAM) (Figure 4B). *Hps5* injected embryos were viable, and displayed mosaic reduced RPE and melanophore melanization into adulthood, as well as a mosaic loss of iridophore pigmentation (Figure 4D).

### Visualizing fluorescent transgenic reporters in casper mutants

Fish embryos are highly transparent and develop externally, allowing for easy visualization of early embryogenesis. Combined with efficient transgene incorporation using Tol2 transgenesis (Kawakami 2005; Erickson *et al.* 2016), sticklebacks represent a powerful system for assaying the activity of developmental enhancers using fluorescent reporter constructs (Erickson *et al.* 2015). However, as fish develop and become more pigmented, imaging becomes extremely difficult, and investigations into late-acting enhancer elements require microdissection. We next sought to test whether the reduced pigmentation of *casper* embryos would allow for easier imaging of enhancer patterns in juvenile fish.

**Figure 5:**
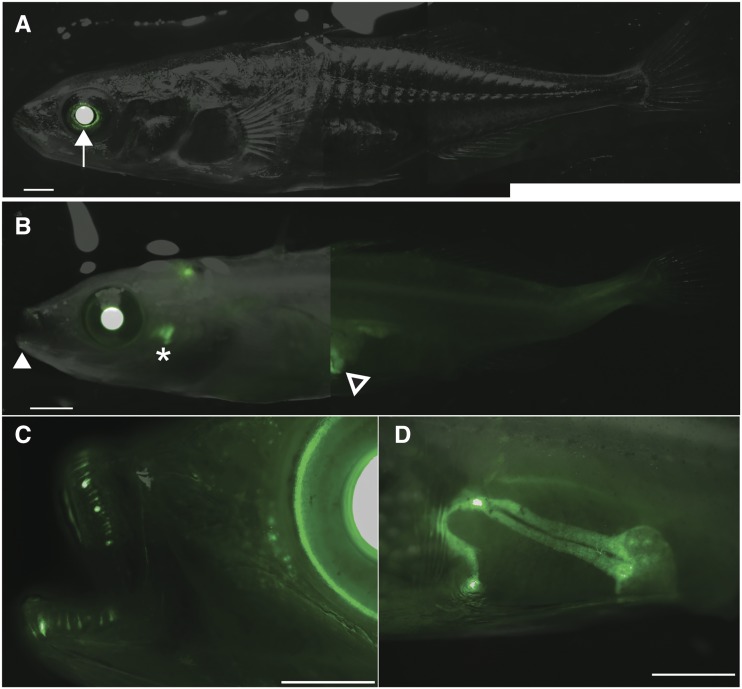
*casper* mutants allow improved visualization of fluorescent reporter genes. (A) A fluorescent GFP reporter of a previously described tooth enhancer (Erickson *et al.* 2015) is only clearly visible in the lenses (white arrow) of wild-type fish. (B) A *casper* sibling with the same stable integration of the fluorescent reporter reveals more readily apparent oral (white filled arrowhead) and pharyngeal teeth (asterisk) domains, and clearly reveals a major previously unreported liver expression domain (black filled arrowhead). (C) GFP reporter activity during oral tooth development and replacement. (D) The left side of the liver expression domain in *casper* fish (right visible in B). Bars, 1 mm.

We crossed a heterozygous *casper* female to a male carrying a single copy of a fluorescent reporter of a previously described 190 bp *Bmp6* enhancer (Erickson *et al.* 2015), previously described to be active in the fins and teeth. Two months postfertilization, wild-type males showed reported green fluorescent protein (GFP) expression in the lens of the eye—a known internal positive control domain of expression of the zebrafish *heat-shock 70-like* (*Hsp70l*) promoter used (Erickson *et al.* 2015)—with other expression domains obscured by pigmented cells (Figure 5A). *casper* mutants carrying the transgene showed similar robust lens expression, but also better revealed other visible juvenile expression domains in oral and pharyngeal teeth, as well as revealed a previously unreported major expression pattern in the liver (Figure 5, B–D).

## Discussion

Here, we present the first stickleback model of Hermansky-Pudlak syndrome, the spontaneous X-linked recessive *casper* phenotype, resulting from a frame-shifting insertion in *Hps5*. *Hps5* mutants display oculocutaneous albinism, with severely decreased pigment in melanophores, iridophores, and erythrophores, though, interestingly, not xanthophores. These pigment phenotypes suggest that pterinosomes in xanthophores develop in a *Hps5*-independent manner, while the melanins, carotenoids, or guanine crystal containing organelles in melanophores, erythrophores, and iridophores develop in a *Hps5*-dependent manner. Pigment was also drastically reduced in the RPE of *Hps5* mutants, suggesting that defects in LRO biogenesis are not restricted to chromatophores, as the RPE does not contain chromatophores (Schraermeyer and Heimann 1999). Additionally, we observed a bleeding phenotype in *casper* mutants, potentially similar to the bleeding diathesis phenotype seen in human *HPS5* mutants (Huizing *et al.* 2004). Overall, these phenotypes agree with the reported phenotypes of mutations in BLOC-2 complex members in other species (Zhang *et al.* 2003; Daly *et al.* 2013; Nakayama *et al.* 2016).

It is unclear why the stickleback *casper* mutation in exon 6 of *Hps5* causes more severe pigmentation phenotypes than the zebrafish *snow white* mutation (Daly *et al.* 2013), which is lethal, unlike the stickleback *Hps5* mutations reported here. Whether more N-terminal mutations within or prior to the WD40 domains also cause lethality in sticklebacks, as previously proposed (Daly *et al.* 2013), could be tested by inducing mutations more N-terminal in *Hps5*.

### Hps5 underlies the casper phenotype

Our mapping-by-sequencing approach using bulk segregant analysis of *casper* mutants revealed a peak genetic signal near *Hps5* on chromosome 19, the stickleback X chromosome. By Sanger sequencing, we found the insertion of a single guanine to a heptaguanine run in the coding frame of *Hps5* in *casper* mutants. This spontaneous insertion might be due to the presence of this homopolymer repeat, as the rate of indel formation is elevated at long homopolymer runs (Montgomery *et al.* 2013), potentially due to polymerase slippage (Levinson and Gutman 1987). Lastly, we showed injection of Cas9 mRNA and *Hps5* guide RNAs resulted in induced mutations in *Hps5* and embryos displaying oculocutaneous albinism phenotypes similar to *casper* mutants, demonstrating *Hps5* disruption underlies the *casper* phenotype. Although our approach using 47 pooled mutants identified a strong candidate gene, future mapping-by-sequencing of other mutations could generate improved genetic resolution by pooling even more mutant DNAs.

### Live imaging of fluorescent reporters in casper embryos

*casper* and *Hps5* mutant embryos are both semitransparent even as adults, unlike their wild-type siblings. This transparency allows for better live imaging of fluorescent transgenic reporters in *Hps5* mutant fish. Imaging a previously characterized enhancer of *Bmp6* (Erickson *et al.* 2015) in *Hps5* mutants, we discovered a major unreported expression domain in the liver. We also found that imaging the previously reported dynamic tooth expression domains (Erickson *et al.* 2015) to be greatly facilitated by the depigmented phenotype of *Hps5* mutants. The viable nature of the *casper* mutation allows for the creation of stable transgenic lines to more easily visualize reporter gene expression, especially at older postembryonic stages. The X-linked nature of the *casper* mutation allows for the recovery of *casper* males from outcrosses to different stable lines within a single generation.

### Genome editing with CRISPR/Cas9

We report the first successful generation of loss-of-function mutations using the CRISPR/Cas9 system in sticklebacks. Coinjection of Cas9 mRNA along with either of two guide RNAs (gRNAs) targeted to exon 6 of *Hps5* resulted in a high frequency (>90%) of embryos with severe or mosaic pigmentation phenotypes. Furthermore, strong pigment phenotypes were seen in both XY males and XX females, implying that Cas9 is able to induce biallelic hits in stickleback embryos. We also see evidence for high efficiency in our single clone Sanger sequencing, as most (8/9) of our sequenced clones contained induced mutations. This high rate suggests that *Hps5* guide RNAs could be used as a marker for other guide RNAs in a co-CRISPR approach (Kim *et al.* 2014; Kane *et al.* 2017), with more albino embryos representing embryos with high levels of nuclear Cas9 activity. As stickleback testes are pigmented, screening for albino testes might further enrich for germline mutations from other coinjected gRNAs.

Injection of even a single gRNA is sufficient to induce large (>25 bp) deletions around the genomic target, similar in size to zebrafish reports, but larger than in human cells (Hwang *et al.* 2013; Paquet *et al.* 2016). Coinjection of two gRNAs resulted in an increase in efficiently edited embryos, suggesting that coinjection results in a high F_0_ mutation induction efficiency and allows phenotypic analysis of F_0_ injected embryos, as we have done here. Furthermore, coinjection of two gRNAs significantly increased the induction of deletions of the intervening sequence between our two *Hps5* guide RNAs, showing that Cas9 can efficiently induce genomic deletions in stickleback embryos. These deletions could have a stronger effect on gene function, and allow for easy and inexpensive genotyping of stable mutants. Additionally, these deletions could be targeted to noncoding DNA such as enhancers, which might not be as sensitive to small deletions as a coding frame. Inducing deletions of regulatory elements will allow functional genetic tests of candidate regulatory elements that underlie evolved changes.

## Supplementary Material

Supplemental material is available online at www.g3journal.org/lookup/suppl/doi:10.1534/g3.117.1125/-/DC1.
