# Supplementary figures and images for: Sequence-Based Mapping and Genome Editing Reveal Mutations in Stickleback Hps5 Cause Oculocutaneous Albinism and the casper Phenotype
Source: G3 (Bethesda). 2017 Jul 26;7(9):3123–31. doi: 10.1534/g3.117.1125 (PMC5592937; doi:10.1534/g3.117.1125)

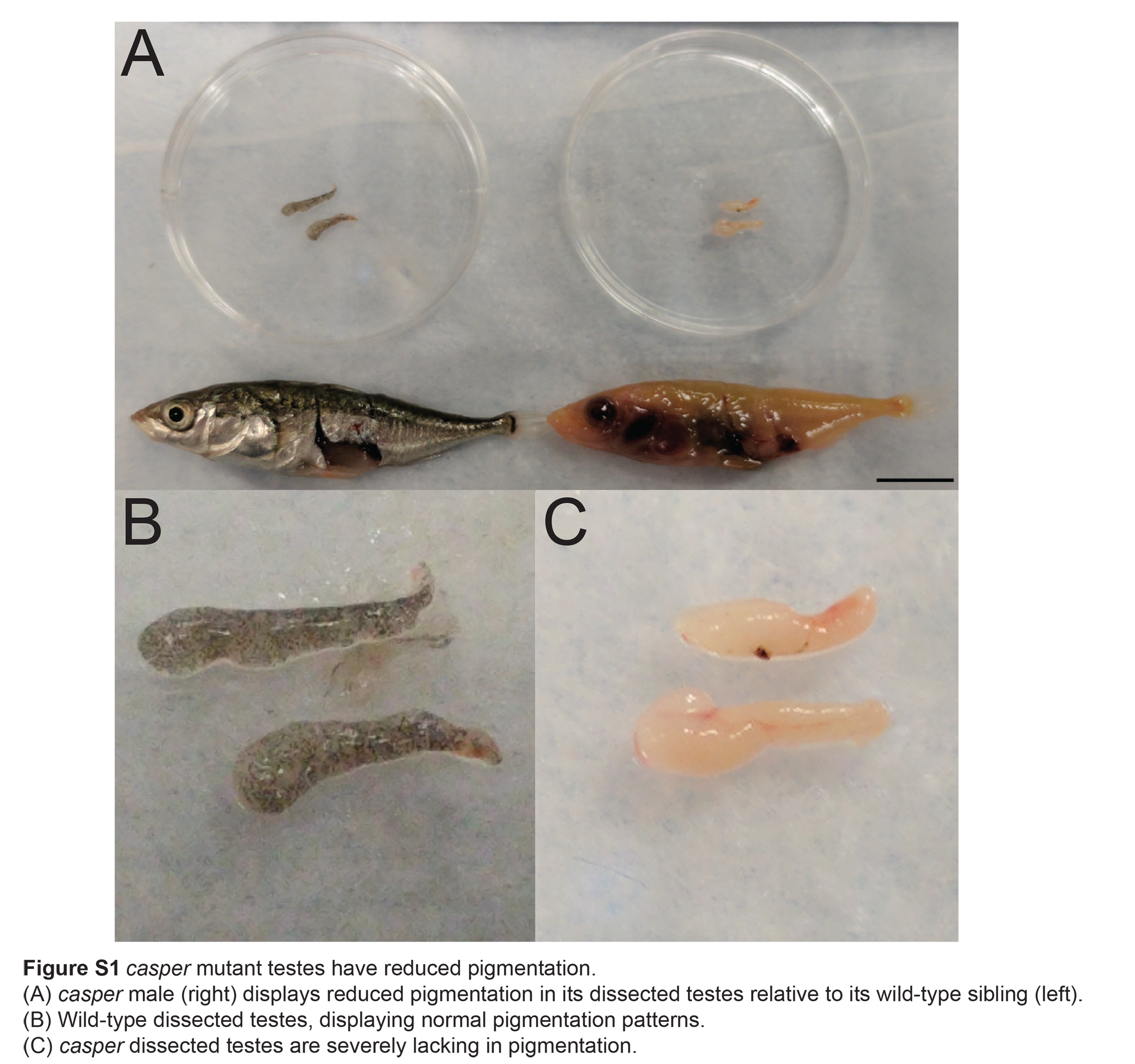

Supplement: Supplementary file 1 [file 3123FigureS1.tif]

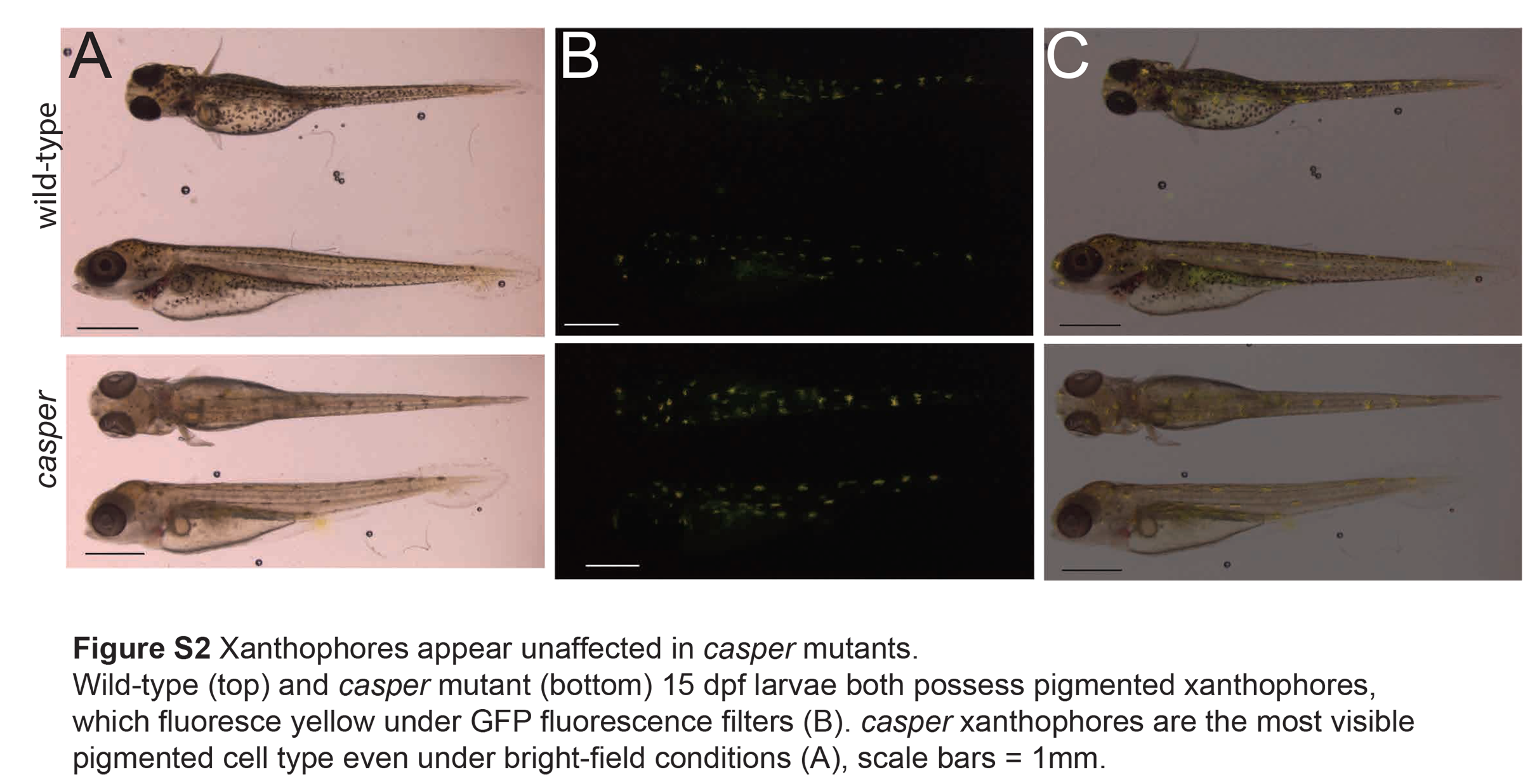

Supplement: Supplementary file 2 [file 3123FigureS2.tif]

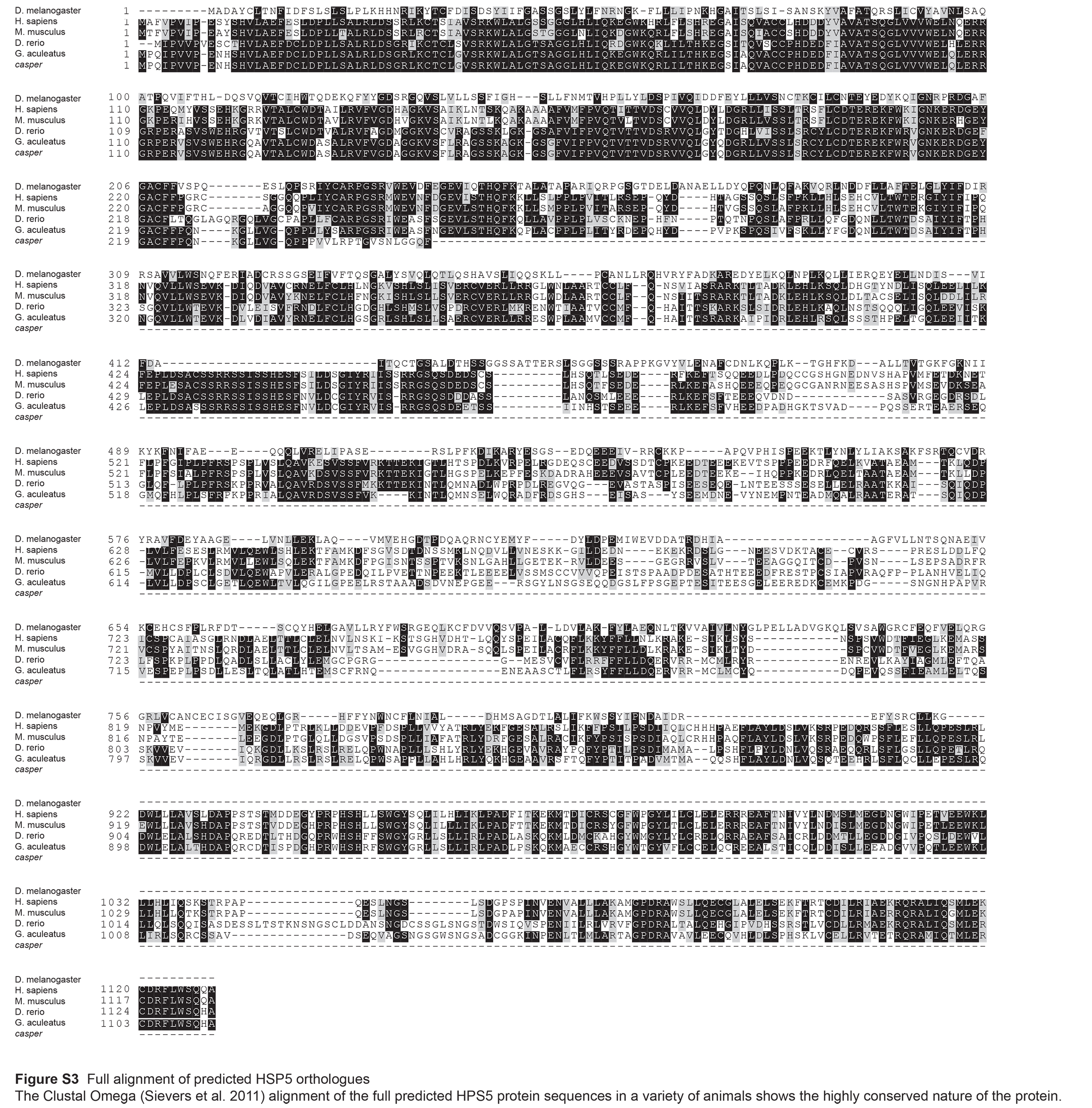

Supplement: Supplementary file 3 [file 3123FigureS3.tif]
