# Supplementary material for: Sequence-Based Mapping and Genome Editing Reveal Mutations in Stickleback Hps5 Cause Oculocutaneous Albinism and the casper Phenotype
Source: G3 (Bethesda). 2017 Jul 26;7(9):3123–31. doi: 10.1534/g3.117.1125 (PMC5592937; doi:10.1534/g3.117.1125)
Supplement: Supplementary file 4 [file 3123TableS1.doc]

**Table S1** Outcrossing female *casper* carriers yields *casper* animals.

| Male population | Male number | casper embryos | wild-type embryos |
| --- | --- | --- | --- |
| RABS | 1 | 19 (23%) | 63 (77%) |
| RABS | 2 | 16 (25%) | 47 (75%) |
| RABS | 3 | 15 (38%) | 24 (62%) |
| RABS | 3 | 22 (23%) | 72 (77%) |
| LITC | 4 | 16 (21%) | 61 (79%) |
| LITC | 4 | 15 (23%) | 49 (77%) |

Each row lists a single cross between a male and a *casper* carrier female. Male population describes the stickleback population of the male parent of the cross (RABS = Rabbit Slough, Alaska, LITC = Little Campbell River, British Columbia). “Male number” is a unique identifier of a single male. Male 3 and 4 sired two different crosses. “*casper* embryos” lists the number of embryos with a *casper* phenotype in the clutch, and “wild-type embryos” lists the number of wild-type embryos in the clutch.
