# Supplementary material for: Sequence-Based Mapping and Genome Editing Reveal Mutations in Stickleback Hps5 Cause Oculocutaneous Albinism and the casper Phenotype
Source: G3 (Bethesda). 2017 Jul 26;7(9):3123–31. doi: 10.1534/g3.117.1125 (PMC5592937; doi:10.1534/g3.117.1125)
Supplement: Supplementary file 5 [file 3123TableS2.doc]

**Table S2** Mapping *casper* by bulk segregant analysis.

| Sample | Total Reads | Mapped Reads | Final Reads | Expected Coverage |
| --- | --- | --- | --- | --- |
| Original casper | 122762025 | 103307721 | 67090400 | 14.5x |
| Wild-type RABS | 117041920 | 96661604 | 63849076 | 13.8x |
| Bulked Segregants | 78373157 | 67075791 | 48780347 | 10.5x |

“Total reads” lists the reads matching the sample barcode. “Mapped reads” indicates the number of reads successfully mapped to a revised assembly of the stickleback reference genome (Jone*s et* al 2012, Glaze*r et a*l. 2015). “Final reads” indicates the number of reads that passed quality filters including removal of PCR duplicates. “Expected Coverage” indicates the expected fold-coverage of each base in the ~463 Mb assembled genome (Jones *et al* 2012) given the number of “Final Reads.”
