# Supplementary material for: Sequence-Based Mapping and Genome Editing Reveal Mutations in Stickleback Hps5 Cause Oculocutaneous Albinism and the casper Phenotype
Source: G3 (Bethesda). 2017 Jul 26;7(9):3123–31. doi: 10.1534/g3.117.1125 (PMC5592937; doi:10.1534/g3.117.1125)
Supplement: Supplementary file 6 [file 3123TableS3.doc]

| Name | Gene | Forward sequence | Reverse sequence | Restriction Cut Sites |
| --- | --- | --- | --- | --- |
| *casper* genotyping | *Hsp5* | CCAGTGGCTGTTTGAACTGA | GGCCACGGTGTTTTTCATTA | NA |
| gRNA validation | *Hps5* | CTTTGGCACTGGGTCGTAAT | GCGAGTACGGAGCATGTTTT | NA |
| gRNA validation cloning | *Hps5* | CGGCctcgagCTTTGGCACTGGGTCGTAAT | CGGCtctagaGCGAGTACGGAGCATGTTTT | *Xho*I, *Xba*I |
| Hps5 guide1 template | NA | GCGTAATACGACTCACTATAGGGAGAGGAGGACAGGCCAGGTTTTAGAGCTAGAAATAGC | AAAGCACCGACTCGGTGCCACTTTTTCAAGTTGATAACGGACTAGCCTTATTTTAACTTGCTATTT CTAGCTCTAAAAC | NA |
| Hps5 guide2 template | NA | GCGTAATACGACTCACTATAGGTCGGGCGGAGTACAACAGGTTTTAGAGCTAGAAATAGC | AAAGCACCGACTCGGTGCCACTTTTTCAAGTTGATAACGGACTAGCCTTATTTTAACTTGCTATTT CTAGCTCTAAAAC | NA |
| Template amplification | NA | GCGTAATACGACTCACTATAG | AAAGCACCGACTCGGTGCCAC | NA |
| Sex determination | NA | CATATTGCTGCTTGTGTGGAAG | GATCCTCCTCGTTCCTACAG | NA |

**Table S3** Sequences of primers used for PCR and creation of sgRNA templates.

“Gene” indicates the corresponding gene in the stickleback genome the primer sequences are derived from, “Forward and reverse sequence” give the sequence of the primers, and “Restriction cut sites” indicates the presence of cut sites for the given enzyme on the 5’ end of the primers. The “Sex determination” primers amplify fragment sizes of 186 bp and 229 bp from the X and Y chromosomes, respectively (Glazer et al., 2014).
