# Supplementary material for: Sequence-Based Mapping and Genome Editing Reveal Mutations in Stickleback Hps5 Cause Oculocutaneous Albinism and the casper Phenotype
Source: G3 (Bethesda). 2017 Jul 26;7(9):3123–31. doi: 10.1534/g3.117.1125 (PMC5592937; doi:10.1534/g3.117.1125)
Supplement: Supplementary file 7 [file 3123TableS4.doc]

**Table S4**  Highly efficient generation of mosaically albino embryos with CRISPR/Cas9.

| Guide RNA | wild-type | mosaic | *casper*-like |
| --- | --- | --- | --- |
| Hps5 guide 1 | 2 | 19 | 5 |
| Hps5 guide 2 | 6 | 25 | 8 |
| Hps5 guide 1 + Hps5 guide 2 | 3 | 14 | 15 |

Each row lists all viable embryos of a single injected clutch. “Guide RNA” indicates whether a single guide or two guides was co-injected along with Cas9 mRNA. “Wild-type” is the number of embryos displaying no visible albino clones. “Mosaic” indicates the number of embryos with albino clones (~1% to ~75% albino). “*casper*-like” indicates the number of embryos that were severely affected (~ > 75% albino).
